# A Review of PMMA Bone Cement and Intra-Cardiac Embolism

**DOI:** 10.3390/ma9100821

**Published:** 2016-10-06

**Authors:** Puneeth Shridhar, Yanfei Chen, Ramzi Khalil, Anton Plakseychuk, Sung Kwon Cho, Bryan Tillman, Prashant N. Kumta, YoungJae Chun

**Affiliations:** 1 Department of Bioengineering, University of Pittsburgh, Pittsburgh, PA 15213, USA; pus8@pitt.edu (P.S.); pkumta@pitt.edu (P.N.K.); 2 Department of Industrial Engineering, University of Pittsburgh, Pittsburgh, PA 15213, USA; yanfeichen@pitt.edu; 3 Division of Cardiology, Allegheny General Hospital, Pittsburgh, PA 15212, USA; rkhalil@ahn.org; 4 Bone and Joint Center at Magee-Women’s Hospital of UPMC, Pittsburgh, PA 15213, USA; plakap@upmc.edu; 5 Department of Mechanical Engineering and Materials Science, University of Pittsburgh, Pittsburgh, PA 15213, USA; skcho@pitt.edu; 6 Division of Vascular Surgery, University of Pittsburgh Medical Center, Pittsburgh, PA 15213, USA; tillmanbw@pitt.edu; 7 McGowan Institute for Regenerative Medicine, Pittsburgh, PA 15219, USA; 8 Department of Chemical and Petroleum Engineering, University of Pittsburgh, Pittsburgh, PA 15213, USA

**Keywords:** PMMA, bone cement, cardiac embolism, cement leakage, viscosity

## Abstract

Percutaneous vertebroplasty procedure is of major importance, given the significantly increasing aging population and the higher number of orthopedic procedures related to vertebral compression fractures. Vertebroplasty is a complex technique involving the injection of polymethylmethacrylate (PMMA) into the compressed vertebral body for mechanical stabilization of the fracture. Our understanding and ability to modify these mechanisms through alterations in cement material is rapidly evolving. However, the rate of cardiac complications secondary to PMMA injection and subsequent cement leakage has increased with time. The following review considers the main effects of PMMA bone cement on the heart, and the extent of influence of the materials on cardiac embolism. Clinically, cement leakage results in life-threatening cardiac injury. The convolution of this outcome through an appropriate balance of complex material properties is highlighted via clinical case reports.

## 1. Introduction

Approximately 700,000 people suffer from vertebral compression fractures in the United States, costing over $1 billion for treatment and management [1]. Reinforcement of vertebral compression fractures with polymethylmethacrylate (PMMA) bone cement through percutaneous vertebroplasty (PVP) was first introduced by Galibert et al. in 1987 [1,2]. PVP was reportedly not conducted in the United States until 1994 [3]. It involves injecting PMMA bone cement into the compressed vertebral body to steady the fracture mechanically [4]. The bone cement popularly used is primarily a two component system composed of a powder PMMA copolymer and a liquid methylmethacrylate (MMA) monomer. This is used because it is a bismethacrylate, thus improving network formation and stability of the cement. The development of this arena has led to many challenges related to the material properties of PMMA. One other procedure that involves the use of PMMA bone cement is kyphoplasty, which involves filling of the collapsed or injured vertebra [5,6]. However, the PVP technique is much more prone to adverse cement leaks than kyphoplasty, because the PMMA is injected in a liquid state in the case of PVP and the cement would flow through the bone path of least resistance [3]. The popularity of the procedure is increasing and it is being used more frequently [7].

PMMA is an inert material which is not reabsorbed in the human body. The polymer achieves 90% of its ultimate strength within one hour of injection [8]. Although alternative materials are available and continue to be created, PMMA remains the material of choice due to its mechanical properties and lower rate of professed complications. An overarching problem associated with PMMA is cement leakage, which may result in both local and global complications [9]. Cement leakage is reported to be as high as 72.5% in metastases and 65% in osteoporotic fractures [10]. Cement leakage may cause local complications (cord compression or nerve root compression) or systemic complications such as pulmonary embolism, cerebral embolism, cardiac embolism, cardiac perforation, renal artery embolism, and acute respiratory distress syndrome [11]. There is also some unknown long-term result in the osteoporotic spine. The damage is more prominent in vital organs such as the lungs and the heart. Even retrograde intra-venous PMMA in femoral nutritional vessels, but without serious side effects from the heart systems, has been documented [12]. In addition, absorption of the PMMA monomer can induce hypotension by virtue of its cardiotoxic and arrythmogenic properties [13].

This review will focus on the mechanisms present at the bone cement interface, related cardiovascular changes in particular, the intra-cardiac embolism, and the various alternatives and solutions that have been described. The paper will also give the reader an idea about important publications on bone cement polymer related cardiac emboli.

## 2. Cement Leakage

One of the significant complications in the reinforcement of vertebral compression fracture is cement leakage, which flows to the cardiac region (i.e., embolization). Here we describe various factors that play a role in cement leakage to the heart. Among all the factors, PMMA viscosity is probably one of the most important determinants dictating cardiac embolism. In addition, advances in mathematical models related to PMMA cement have been discussed in detail.

### 2.1. Factors Affecting Cement Leakage

Several factors related to cement leakage include: bone permeability, marrow viscosity, bone porosity, size of the injection cavity, diameter of the leakage path, bone pore size, and cement viscosity [15]. Among these factors, the cement viscosity is the only parameter not determined by the bone structure, indicating that the bone cement leakage can be controlled by adjusting the cement viscosity. Therefore, PMMA with low and medium viscosity has been identified as an important risk factor for the development of cement leakage along with bone porosity. High-viscosity PMMA, on the other hand, effectively stabilizes vertebral compression fractures, while minimizing the risk of cement leakage and associated complications in vitro [14,15]. Subsequently, decompressed PVP may result in reduced cement leakage [16]. Nieuwenhuijse et al. performed a detailed analysis of potential risk factors for the occurrence of cement leakage, and fracture severity and PMMA bone cement viscosity were identified as two strong independent predictors in general [17]. Lador et al. studied leakage patterns that understood the points and patterns of cement extravasation in 23 human vertebrae. This study showed that the most common type of leakage, classified as severe, occured through small breaches in the cortex due to anterior blood vessels. Severe leakage occurred in 83% of the samples, and suggested monitoring procedures during injection to avoid complications and to minimize possible life-threatening risks to the patients [18]. The leakage to the nearby vasculature may reach distant locations of the body, such as the heart, and result in cardiac embolism [19].

The use of high-viscosity cement seems to stabilize cement flow. However, the forces required for the high-viscosity cement delivery are significantly higher and may approach or even exceed the human physical limit of injection forces. Higher injection forces may also result in poor filing of the cement [20]. Another possibly detrimental effect due to the use of high-viscosity cement would be the increase in the extravasation of bone marrow into the cardiovascular system.

### 2.2. PMMA Viscosity Behavior

**Figure 1:**
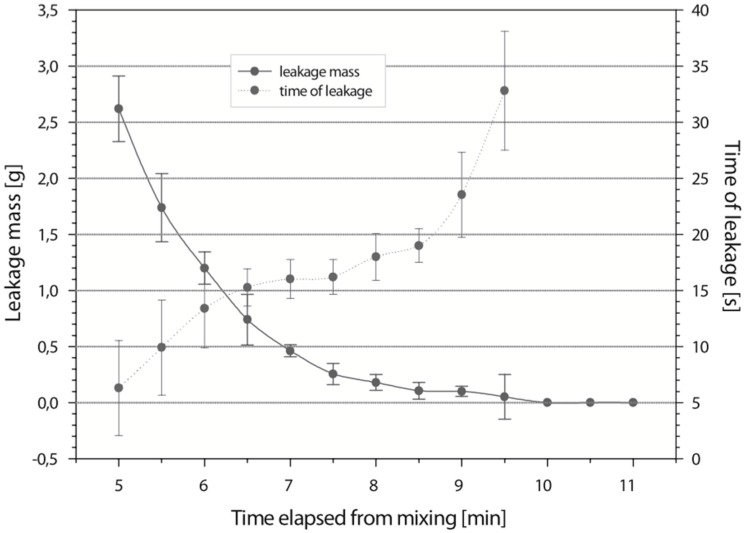
Depiction of leaked cement mass and time when leakage occurred with elapsed time. Adapted from [21], with permission from © 2006 Wolters Kluwer Health, Inc..

Viscosity is probably the most important material property responsible for cement leakage. An ideal bone cement, when injected, should stabilize vertebral compression fractures. An increase in the viscosity of PMMA cement is associated with a fast polymerization process. This actively relates with a higher released temperature and a shorter setting time (Figure 1) [21]. Additionally, the viscosity steadily increases during the PMMA polymerization process. The storage and loss moduli also increase over time as the PMMA polymerization progresses [22]. In addition, the bone cement should be injected at the latest time point possible in order to prevent leakage and extravasation. This ultimately minimizes the risk of cement leakage and related complications.

Hydrogen peroxide, used as hemostatic agent in arthroplasty, has been shown to adversely affect the material properties of PMMA [19]. Properties of PMMA cements such as time and shear rate are essential for examining the cement flow behavior [23]. In addition, a greater chance of embolism and a reduction in arterial oxygenation resulting in circulatory failure was observed in the use of hand-mixed PMMA when compared to the use of vacuum-mixed PMMA [24]. It has been further reported that drilling a hole with and without vacuum can potentially reduce the intraosseous pressure and, thus, reduce the risk of emboli [22]. Factors such as viscosity, permeability in cancellous bone, and biomechanical strength of the mixture also play a crucial role [14].

The rheological properties are important factors influencing the cement flows and they may play a part in the formation of the pores in the cement during polymerization. These pores are postulated to act as sites for the initiation of cracks which contribute to the aseptic loosening of the prosthesis [25]. During the bone cement curing, initially the rise in viscosity is largely due to the swelling of the polymer particles in the monomer, while polymerization of the monomer also contributes and finally dominates the rise in viscosity at later times, suggesting a strong temperature dependence of the viscosity-time profiles [26]. Therefore, PMMA bone cement viscosity is not a constant and it has two different characteristics: (a) rheopectic (where the viscosity rises with time) and (b) pseudoplastic (where the viscosity drops with shear rate). In order to characterize the time-dependent permeability of the bone cement, the time and shear rate dependent viscosity was captured by the following power law by Baroud et al. [27]:

(1) $$\eta =\left[a\left(\frac{t}{t_{s}}\right)+b\right]{\left(\frac{\gamma }{\gamma_{s}}\right)}^{c\left(t/t_{s}\right)+d}$$

where *η* is the viscosity, *γ* is the shear rate, *t_s_* and *γ_s_* are the characteristic time and shear rates, respectively, and *a*, *b*, *c*, *d* are the viscosity material parameters. This rheological model of PMMA bone cements was implemented in ANSYS (Finite Element package) and the agreement between the analytical and numerical solutions confirmed that the proposed model appropriately captured both the rheopectic and pseudoplastic behaviors [24]. The finite element analysis indicated a logarithmic increase of the injection pressure due to the non-uniform viscosity profile in the cannula. It was also noted that the non-linear increase almost doubled over a period of two minutes. This model could be further implemented to predict the cement flow behavior.

### 2.3. PMMA Cement Based Mathematical Model

A theoretical model was proposed by Bohner et al. to analyze the distribution of a PMMA cement after its injection into a porous structure [28]. The calculations were based on two rheological laws: the law of Hagen-Poiseuille describing the flow in a cylindrical tube and the law of Darcy describing the fluid flow through a porous media by assuming that the path of least resistance is cylindrical and that the cement can only extravasate if it pushes the marrow out of the way when the cement is injected into an osteoporotic vertebral body. The ratio between the augmentation pressure (the pressure required to expand the cement spherically) and the extravasation pressure (pressure required to inject the cement into the path of least resistance) defined as the risk factor for extravasation can be calculated by:

(2) $$\lambda =\frac{\Delta P_{a}}{\Delta P_{e}}=\frac{D_{e}^{4}}{512pK}f\left(\mu_{c},\mu_{m},t,R_{0},L_{e}\right)$$

where *D_e_* is the diameter of the path of least resistance, *p* is a dimensionless parameter of the matrix porosity, *K* is the matrix permeability, *μ_c_* is the cement viscosity, *μ_m_* is the marrow viscosity, *R*_0_ is the radius of the cavity at the injection point, *L_e_* is the length of the path of least resistance, and *f*(*μ_c_*, *μ_m_*, *t*, *R*_0_, *L_e_*) is a function of the parameters within the brackets. Extravasation occurs when the risk factor is larger than 1.

In order to account for the non-Newtonian nature of curing PMMA in a simulation of PMMA injected through a cannula to improve quantitative accuracy, Lian et al. developed a biochemical model for PMMA injection by approximating the cancellous bone trabecular network as a branching-pipe network [29]. The overall pressure drop across the branch segment that is represented as a conical pipe with radii *R*_1_ and *R*_2_ at its ends can be derived as [29]:

(3) $$-\Delta P=\frac{8\mu LQ}{\pi }\left[\frac{1}{3}\left(\frac{1}{R_{1}R_{2}^{3}}+\frac{1}{R_{1}^{2}R_{2}^{2}}+\frac{1}{R_{1}^{3}R_{2}}\right)\right]$$

and the effective wall shear-rate magnitude is then

(4) $${\left|\dot{r}\right|}_{wall}=\frac{4Q}{\pi {\left(\sqrt{R_{1}R_{2}}\right)}^{3}}$$

where *Q* is the flow rate, *μ* is the dynamic viscosity of PMMA, *R* is the cannula radius, and *L* is the cannula length.

This computational model approach demonstrated the potential of simulating PMMA injection into cancellous bone during percutaneous vertebroplasty. This model employed the Hagen–Poiseuille law to predict the pressure drop across a delivery cannula with viscoelastic changes of curing PMMA modeled via a time and shear-rate dependent power law. The power law that was derived based on dynamic rheological testing of curing PMMA samples was fitted with experimental data. In conjunction with a branching-pipe geometrical model, the method helped with quick estimation of the overall injection pressure, and, hence, the reaction force during manual PMMA injection (Table 1). For real-time simulations, a challenge is to “refresh” (or update) the rendered images and computed forces every 20 and 2 ms for realistic visual and haptic force feedback, respectively [29].

### 2.4. Intra-Cardiac Embolism Leading to Cardiovascular Deterioration

Intra-cardiac embolism (ICE) secondary to PMMA leakage could be an incidental finding or it may appear during the procedure, immediately following the procedure, during hospital recovery, or even manifest as a long term complication (ranging from days to years) (Table 2, [30,31,32,33,34,35,36,37,38,39,40,41,42,43,44,45,46,47,48,49,50,51]). The clinical significance of cement emboli as an incidental finding on chest radiographs may not be ignored due to their known long-term sequelae. Early detection and immediate management should be the key despite the absence of clinical symptoms. When emboli are discovered incidentally on a conventional chest X-ray, the heterogeneity in shape and pattern make it extremely difficult to arrive at a proper diagnosis. In such a situation, further evaluation by transthoracic echocardiography, transesophageal echocardiography, coronary tomography scan, and cardiac magnetic resonance imaging is instrumental in arriving at the final diagnosis. These diagnostic aids are also helpful in the evaluation of pericardial effusion and valvular defects. It is extremely important to rule out conditions such as patent foramen ovale or atrial septal defect (ASD) with the help of a bubble study, as there is a higher probability of paradoxical embolism related to tiny mobile fragments leading to stroke which may result in the wrong diagnosis [47].

Adding hydroxyapatite (HA) to PMMA cement to reduce the quantity of barium, which is used as a radiopacifier, may aggravate cardiovascular deterioration in the event of cement embolism by the activation of coagulation. Acute cardiovascular consequences of considerable PMMA leaks (2 mL) may not be severe in persons with a healthy cardiopulmonary system. Subsequent addition of hydroxyapatite (10%) to PMMA cement did not result in more severe cardiovascular changes [52]. It is possible that thromboembolism may also aggravate cardiovascular deterioration after PMMA embolism. In addition, it would be impossible to control the quantity of embolized cement [53].

## 3. Alternatives to PMMA

### 3.1. Calcium Phosphate and Hydroxyapatite

Over time, many varieties of injectable materials have been proposed for use in order to reduce cement leakages [54]. Calcium phosphate (CP) cement has been used as a clinical substitute to PMMA. rhBMP-2/CP, an osteoinductive and biodegradable material, is another candidate that may also be an alternative to PMMA, in order to achieve biostabilization in a vertebroplasty [55]. It has been demonstrated in animal experiments that fragmentation of calcium phosphate cement yields more emboli, especially microemboli, resulting in more severe cardiovascular deterioration when compared to PMMA; this was confirmed by CT scanning of postmortem lungs [56]. Even though CP is biocompatible, a major drawback is that it rapidly decays when in contact with blood or physiological fluids. The compressive strength is very similar to PMMA and its isothermic properties during the setting phase prevents heat related tissue damage [57]. Additionally, it is known that the severity of pulmonary hypertension after embolism is related to the size and number of emboli [58].

Meanwhile, vertebroplasty with CP yields better clinical and radiological results than conservative treatments for primary vertebral fractures, with the exception of some intraoperative complications, such as leakage and embolism [56]. Another concern raised is that the use of self-setting calcium orthophosphate formulations might aggravate cardiovascular deterioration in the event of pulmonary cement embolism by stimulating coagulation [57]. One other disadvantage of CP is its poor injectability. Liquid–solid phase split-up has been noted in commercial formulations [58]. The addition of calcium phosphate fillers into PMMA bone cement has been reported to be detrimental to cement handling and mechanical performance [22].

Hydroxyapatite (HA) has been explored as an alternative. The properties of bone conductivity and the absence of exotherm of hydroxyapatite-forming materials make them an attractive alternative to PMMA cements [4]. Thus, we believe that the use of HA improved cement is worth it because of its reduced possibility of causing intra-cardiac and distant embolism when compared to the use of PMMA cement alone. A paired-design study identified some indirect but mostly insignificant differences in immediate biomechanical fixation of pedicle screws augmented with the Sr-HA cement compared with the PMMA cement [66].

### 3.2. Radio-Opacification

Proper opacification is essential for fluoroscopic monitoring of cement injection to prevent extravasation and, thus, the potential complication of pulmonary embolism [59,60]. The addition of nanoparticle radiopacifiers, such as barium sulfate and zirconium dioxide, improve osteoblast adhesion rather than plain PMMA bone cement. Barium sulfate improves the visibility of the PMMA since it has a higher atomic number and attenuates the X-rays. Unfortunately, some detrimental effects of these radiopaque agents on the mechanical behavior of PMMA have been observed [61]. Hernandez et al. showed that a PMMA cement with 10% w/w barium sulfate has a similar viscosity-time curve, but a much earlier onset of viscosity rise compared to the same cement with no radiopacifier [62]. This highlights the effect of varying the radiopacifier composition on cement viscosity, and thus the injection behavior of that cement suspension.

There are adverse effects on injectability, viscosity profile, setting time, mechanical properties of the cement, and bone resorption. Altogether, radio-opacifiers are considered to be beneficial depending on their type and concentration. In order to overcome these issues, PMMA microspheres in which gold particles are embedded and its monomer is the same as that used in commercial cements for vertebroplasty have been attempted [63].

### 3.3. Orthocomp™ and Hydroxyapatite

Orthocomp™ is composite material with a matrix of Bis-phenol glycidyl dimethacrylate (BisGMA), Bis-phenol ethoxy dimethacrylate (BisEMA), and triethyleneglycol dimethacrylate (TEGDMA) [4]. It is biocompatible, has a lower setting exotherm, and good material properties. In a study comparing compressive moduli, Orthocomp™ exhibited a modulus almost twice that of the PMMA cements. In fact, Jasper et al. suggested that a higher modulus may translate to better mechanical stabilization, resulting in a lower use of Orthocomp™ compared to PMMA cement [55]. Similarly, for compressive yield strength and ultimate compressive strength, PMMA cements ranged from 50 ± 73 MPa and from 53 ± 80 MPa, respectively, but Orthocomp™ exhibited strength values 2–3 times those values [64].

### 3.4. Injection Device and Viscometer

Various optimization tools have also been utilized. The impact of the injection device and viscometer in controlling cement leakage has been suggested. Gisep’s study showed a strong correlation of the PMMA viscosity during setting to the injection forces through vertebroplasty injection devices [67,68]. There was a significant difference in injection forces between the in vitro injections in room temperature or in a simulated body temperature setting. The viscometer may potentially have a role in enhancing the safety of percutaneous vertebroplasty procedures. Transpedical body augmentors have been used to try and prevent body recollapse, as well as PMMA in the short term, which when used with bone grafts theoretically allows for potential fracture healing in the long term.

### 3.5. Drug Delivery System, Porous PMMA, and Cementless Procedure

The drug delivery system releasing Vancomycin, an antibiotic, from bone cement is controlled according to Higuchi’s theory. Imperfect polymerization of the polymer could cause the monomer to leak and therefore change the matrix structure. Hence, it could affect the release of antibiotics from bone cement beads. One must prepare cement beads properly, taking into consideration Higuchi’s equation in order to control release by adjusting the amount of drug in the beads and the diameter of the cement device [69].

The particle release of porous PMMA cements during curing has been studied, because the release of powder ingredients obtained from porous PMMA can theoretically cause negative effects such as embolism. The invention of porous PMMA, in order to make regular PMMA cement more compliant with cancellous bone, while initially promising, remains questionable [70]. The risk of cement leakage can also be decreased by using viscoplastic bone cement due to its lower infiltration depth [71]. However, no direct indicators have been identified up to now that can predict cement leakage to prove superiority of the viscoplastic cement. Further long-term outcome studies comparing cemented to cementless arthroplasty are still needed [24,36]. The possibility of cementless vertebroplasty remains unknown. Saleh and his colleagues have indicated cementless fixation may have a positive stint in the younger population [22].

## 4. Case Report

PMMA cement cardiac embolism is a potentially serious complication following vertebroplasty and kyphoplasty. The frequency of pulmonary cement emboli following percutaneous vertebroplasty varies from 4.6% to 26.9% [72,73,74,75]. The risk of ICE remains unknown. ICE may be an isolated finding or it can co-present with pulmonary emboli. Common chief complaints are chest pain, dyspnea, and syncope. Presentation may be acute, sub-acute, or chronic. Cardiac perforation, acute valvular damage, and paradoxical cerebral embolism are the most dreadful complications. Surgery is the most common management approach. Percutaneous methods involving snaring and the insertion of an inferior vena cava filter have been successful. Conservative medical therapy has also been attempted. We present a case of left ventricular PMMA cement emboli via a secundum ASD causing acute torrential mitral regurgitation (MR). Our anecdotal case illustrates the need for close monitoring of patients undergoing percutaneous vertebroplasty and kyphoplasty. We also emphasize the importance of the treatment of any intra-cardiac cement emboli (ICE) because of its capability of causing serious complications.

A 61-year-old woman with a history of ovarian carcinoma on chemotherapy and vertebral osteoporotic compression fractures presented with acute onset shortness of breath. She was undergoing evaluation for prolonged back pain. Magnetic resonance imaging (MRI) showed compression fractures at the T11, L1, and L2 vertebrae with no significant retropulsion. In view of her persistent pain and the failure of conservative management, she underwent vertebroplasty of the T11, L1, and L2 vertebrae using PMMA cement mixed with barium at an outside hospital. There was a small amount of cement extravasation into the paraspinal veins and prevertebral veins that was noted during the procedure.

**Figure 2:**
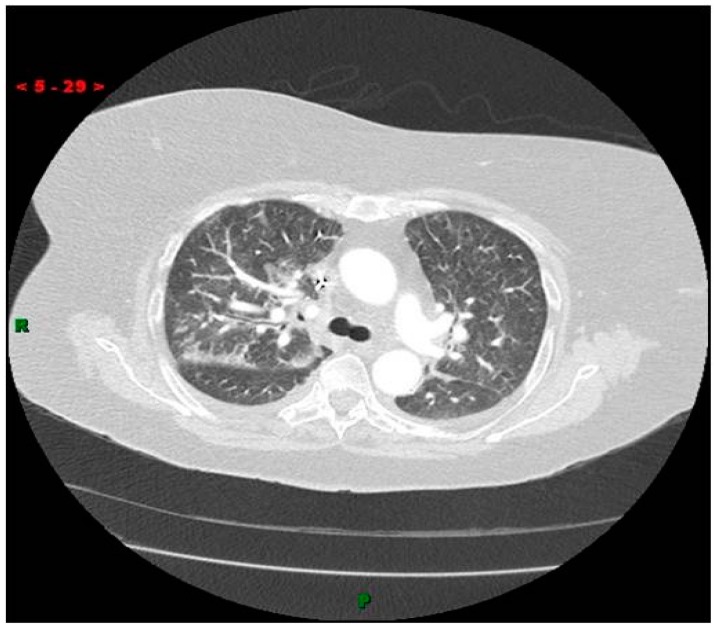
Computed Tomography (CT) scan of the thorax showing numerous pulmonary radiodensities with suspected cardiac radio-densities.

**Figure 3:**
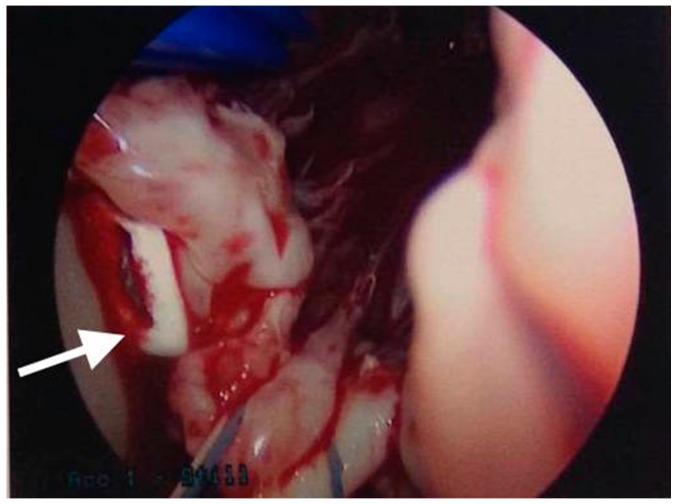
Intra-operative image showing linear, smooth elongated PMMA cement in the left ventricle (white arrow).

Eight hours after the procedure, the patient complained of shortness of breath. She was intubated due to profound hypoxia. Chest X-ray and a Computed Tomography (CT) thorax scan showed multiple dense pulmonary radio-densities along with the presence of cardiac radio-densities (Figure 2). Transthoracic echocardiography (TTE) and transesophageal echocardiogram (TEE) showed linear, smooth, elongated wire-like echo-densities in the left ventricle, a secundum ASD, and severe MR with flail A2 scallop. Fluoroscopy during coronary angiography confirmed the presence of foreign material in the pulmonary vasculature whose radio-density was identical to the material in the left ventricle. An intra-aortic balloon pump was also placed. Endovascular retrieval of the cement fragment was considered. However, given the presence of severe mitral regurgitation, confirmed ASD, and more than one ICE, the decision was made to approach surgically. The patient was taken to the operating room. She was placed on cardiopulmonary bypass. An ostium secundum atrial septal defect measuring 1.5 cm in diameter was present. There was severe destruction of the mitral valve apparatus with rupture of all but one chordae to the anterior mitral leaflet. A few ruptured chordae to the posterior mitral leaflet were also noted. Under thoracoscopic guidance, the two elongated tan white fragments, one measuring 3 cm and the other 1.5 cm in length, which were wedged into the trabeculae of the left ventricle were removed (Figure 3). A 29 mm St. Jude Epic porcine valve was implanted and the ASD was closed with 3-0 Prolene sutures. No residual MR was noted with intra-operative TEE. Her hospital stay remained uneventful and she was discharged to a rehabilitation facility after 7 days.

The frequency of ICE remains unknown. Only a few isolated case reports exist in the literature. ICE is attributed to the passage of the leaked PMMA from the perivertebral veins into the azygos vein and then onwards to the inferior vena cava and finally to the right cardiac chambers [30]. In case of the presence of patent foramen ovale (PFO) or ostium secundum atrial septal defect (ASD), PMMA may cross over to the left atrium and left ventricle and could result in paradoxical embolism. Llanos et al. reported the cement fragment impacted in the inter-atrial septum and protruded into the left atrium [32]. We described the presence of PMMA cement in the left ventricular cavity which embolized through a large ASD and caused the rupture of chordae, consequently resulting in severe mitral regurgitation. There is one report of tricuspid regurgitation caused by an embolic cement fragment [32]. Otherwise, there are reviews of cement pulmonary embolism without any reviews about ICE even though there are a considerable number of instances (Table 2) [30,31,32,33,34,35,36,37,38,39,40,41,42,43,44,45,46,47,48,49,50,51].

Some authors have reported the use of a preinjection venogram to decrease the incidence of pulmonary embolism, and the injection of sclerosing agents into the vertebral body before vertebroplasty has also been suggested to close venous channels [74,75,76]. Low viscosity PMMA cement has also been suggested, but it is not devoid of cardiac embolism. We strongly believe that performing multiple sitting vertebroplasties can decrease the rate of embolic complications.

The treatment for symptomatic ICE is surgical retrieval. It is extremely useful if the bone cement is densely adhered to the adjoining cardiac wall or if it is free floating in the pericardial space [30,31]. In the case of cardiac perforation, immediate pericardiocentesis followed by surgery is required. Valvular conditions associated with cement emboli may require additional valve replacement surgeries depending on the severity of the regurgitation.

In select cases, percutaneous removal by snare catheter and/or trapping of the embolic fragments by Greenfield filter [40] (especially in the case of IVC embolus) may be attempted with extreme caution. More conservative management with anticoagulants should be reserved for peripheral emboli.

## 5. Conclusions

In this paper, we have reviewed the aspects of PMMA material properties that are relevant to orthopedic and cardiovascular applications. It is important to note that cement leakage is asymptomatic in most situations. PMMA viscosity regulated by density and particle size is one of the important determinants in the formation of the cardiac emboli that may have life threatening implications. Additionally, avoiding excessive PMMA injection and excessive pressure during injection may play a role in prevention [11]. Recently, the role of injectable hydrogels has been explored in vertebroplasty [77]. Future work should attempt to understand its favorable impact on the reduction of cardiac as well as non-cardiac embolism.

However, despite large investments into engineering research, developments remain limited mostly as a result of the lack of availability of the perfect candidate for bone cement. Long term complications can be devastating due to PMMA presence in the coronary arteries or in the chambers of the heart. The careful evaluation and search for the ideal bone cement systems and methods is crucial for important orthopedic procedures such as vertebroplasty and kyphoplasties.

## Figures and Tables

**Table 1:** Mean ± standard deviations for the measured and calculated parameters: cement viscosity at injection, injection forces, injection speed, and normalized injection force. Adapted from [7], with permission from © 2009 Springer.

Differnt Groups	Cement Viscosity at Injection (Pa s)	Injection Force (N)	Injection Speed (mm/s)	Normalized Injection Force (N/Pa mm)
Control group	49.6 ± 13.4	64.6 ± 37.2	6.7 ± 5.4	0.42 ± 0.723
Lavage group	44.6 ± 10.3	54.5 ± 33.9	9.3 ± 3.5	0.16 ± 0.15
Mann-Whitney test/*p* values	0.401	0.361	0.02	0.73
Homogeneity in control group/*p* values	0.00.	0.007	0.12	0.000
Homogeneity in lavage group/*p* values	0.737	0.161	0.981	0.000

**Table 2:** Literature review on Intra Cardiac Emboli (ICE) located in various regions of the heart including the Right Ventricle (RV), Right Atrium (RA), Left Atrium (LA), and Inferior Vena Cava (IVC).

Caption	Procedure	Indication	Time of Event	Symptoms	Location of Embolus	Treatment	Complication
Pannirselvam V	Vertebroplasty	Multiple myeloma	9 months	Syncope	RA	Medical	-
Berthoud B	Kyphoplasty	Osteolytic Metastasis	-	-	RA	-	Pericardial Tamponade
Arnáiz-García ME	Vertebroplasty	Traumatic Vertebral body fracture	During procedure	Hypotension, Respiratory distress	RV	Surgery	-
Moon MH	Vertebroplasty	Compression Fracture	5 years	Chest pain, Fever	RV	Surgery	Pericardial Effusion
Gosev	Kyphoplasty	Compression Fracture	10 days		RV	-	pericardial Effusion
Llanos RA	Vertebroplasty	Fusion, fracture	2 months	Chest pain, dyspnea	LA protuding through atrial septum	Surgery	-
Tran I	Balloon Kyphoplasty	-	1 day	Chest pain, dyspnea	RV	Snare catheter	Pericardial Tamponade
Lee JS	Vertebroplasty	Compression fracture	6 years	Dyspnea	RA, RV and the RV outflow track	Medical	-
Agko M	Kyphoplasty	Fusion, Fracture	During procedure	None	IVC	Greenfield filter	-
Cadeddu C	Vertebroplasty	Compression fracture	2 years	Accidental finding	RV, RA	-	-
Braiteh F	Vertebroplasty	Compression fracture	5 months	Chest pain, Palpitation	RV, RA	Snare	-
Caynak B	Vertebroplasty	Possible Fracture	2 months	Dyspnea	Right side (pericaridal space)	Surgery	Pericardial Tamponade
Son KH	Vertebroplasty	-	10 days	Chest pain, Dyspnea	RA, RV, Pericardial space (right side)	Surgery	Cardiac perforation, Triscupid regurgitation
Lim KJ	Vertebroplasty	Compression fracture	5 years	Dyspnea Leg edema	RA	Surgery	-
Lim SH	Vertebroplasty	Compression fracture, Multiple myeloma, osteolytic metastases	-	Chest pain, dyspnea	RV	Surgery	Multiple cardiac perforation
Scroop R	Vertebroplasty	post-trauma osteoporosis	During procedure	Hypotension	Cerebral Embolism	-	Patent foramen ovale
Kim SY	Vertebroplasty	-	7 days	Chest pain	RA, RV	Surgery	Cardiac perforation
